# Regulation of MicroRNAs-Mediated Autophagic Flux: A New Regulatory Avenue for Neurodegenerative Diseases With Focus on Prion Diseases

**DOI:** 10.3389/fnagi.2018.00139

**Published:** 2018-05-15

**Authors:** Syed Zahid Ali Shah, Deming Zhao, Tariq Hussain, Naveed Sabir, Lifeng Yang

**Affiliations:** National Animal Transmissible Spongiform Encephalopathy Laboratory and Key Laboratory of Animal Epidemiology and Zoonosis of Ministry of Agriculture, College of Veterinary Medicine and State Key Laboratory of Agrobiotechnology, China Agricultural University, Beijing, China

**Keywords:** prion diseases, microRNAs (miRNAs), biological processes, biomarkers, autophagic flux, Alzheimer’s disease, Parkinson’s disease, therapeutic interventions

## Abstract

Prion diseases are fatal neurological disorders affecting various mammalian species including humans. Lack of proper diagnostic tools and non-availability of therapeutic remedies are hindering the control strategies for prion diseases. MicroRNAs (miRNAs) are abundant endogenous short non-coding essential RNA molecules that negatively regulate the target genes after transcription. Several biological processes depend on miRNAs, and altered profiles of these miRNAs are potential biomarkers for various neurodegenerative diseases, including prion diseases. Autophagic flux degrades the misfolded prion proteins to reduce chronic endoplasmic reticulum stress and enhance cell survival. Recent evidence suggests that specific miRNAs target and regulate the autophagic mechanism, which is critical for alleviating cellular stress. miRNAs-mediated regulation of these specific proteins involved in the autophagy represents a new target with highly significant therapeutic prospects. Here, we will briefly describe the biology of miRNAs, the use of miRNAs as potential biomarkers with their credibility, the regulatory mechanism of miRNAs in major neurodegenerative diseases such as Alzheimer’s, Parkinson’s, and prion diseases, degradation pathways for aggregated prion proteins, the role of autophagy in prion diseases. Finally, we will discuss the miRNAs-modulated autophagic flux in neurodegenerative diseases and employ them as potential therapeutic intervention strategy in prion diseases.

## Introduction

Prion diseases also known as transmissible spongiform encephalopathies (TSEs) are a family of neurodegenerative diseases affecting several mammalian species including humans. The most commonly observed prion diseases of animals include bovine spongiform encephalopathies (BSE) in cattle, scrapie disease in sheep and goats, chronic wasting disease in elk and wild deer. Human populations are equally affected with TSEs, including kuru, Creutzfeldt-Jakob disease (CJD), Gerstmann–Straussler–Scheinker syndrome (GSS) and Fatal Familial Insomnia (FFI) (Nakagaki et al., 2013; Puig et al., 2016). The most critical step in prion propagation is the conversion of normal cellular prion protein (PrP^c^) into an abnormal and protease-resistant, misfolded and aggregated isoform termed PrP^Sc^ (Prusiner, 1998). The misfolded PrP^Sc^ is abundantly rich in beta-sheet conformation as compared to its predecessor PrP^c^, which is alpha-helical in structure (Pan et al., 1993; Mukherjee and Soto, 2011; Yunsheng et al., 2015; Song and Kim, 2017). Initial neuroinflammation leads to severe neurodegeneration in prion diseases due to accumulation and aggregation of PrP^Sc^ in the brains of affected individuals (Moreno et al., 2013; Shah et al., 2017a,b,c). The exact molecular mechanisms leading to neuronal inflammation and death are still unclear, and hence there is no cure available for this fatal neurodegenerative disorder (Moreno et al., 2013).

A cell is a fundamental unit of all living organisms, and it runs several biochemical and physiological reactions with high precision and accuracy. There are several regulatory components and pathways of these reactions, which tightly regulate cellular homeostasis. Researchers focus on cellular biomarkers for the study of these highly complicated cellular reactions. These biomarkers are not only critical for providing key information about the standard cellular functions, but they also provide information regarding the pathogenic conditions arising from the endogenous or exogenous stimulus. Modern molecular techniques such as microRNA (miRNA) array-analysis, Northern-analysis, quantitative RT-PCR analysis, and RNA-sequencing-based analyses have uncovered a small group of inducible, pathogenic miRNAs, which are significantly upregulated in the degenerating central nervous system of affected individuals. These miRNAs are involved in the dysregulated expression of disease-relevant messenger RNA (mRNA) targets (Lukiw et al., 2011; Yaghmoor et al., 2014; Zhao et al., 2015a,b; Bhattacharjee et al., 2016; Clement et al., 2016).

The miRNAs are crucial regulators at the post-transcriptional level of genes, and they take an active part in various normal biological and numerous pathological processes involved in these neurodegenerative diseases (Szafranski et al., 2015). miRNAs constitute of 21–23 nucleotides in length. At post-transcriptional level, some important genes are expressed under the influence of these miRNAs (Lee et al., 1993). Pathogenesis of neurodegenerative diseases is a very complex process, and more than 2000 miRNAs have been identified in humans. Large numbers of these miRNAs are directly involved in the disease progression, which gives them the unique role of potential biomarkers in many diseases including Alzheimer’s disease (AD), Parkinson’s disease (PD) and prion diseases (Lee et al., 1993; Cissell et al., 2008; Liu et al., 2008). Several brain pathologists have reported variation in the profile of miRNAs. Some important genes are targeted by these miRNAs in various neurodegenerative disorders (Eacker et al., 2009; Provost, 2010; Sonntag, 2010). Alteration in the synaptic structural plasticity is the earliest hallmark of all major neurodegenerative diseases. Boese et al. (2016) recently demonstrated that during preclinical stage of prion disease, miRNAs enriched in synaptoneurosomes including miRNA-124a-3p, miRNA-136-5p and miRNA-376a-3p were elevated. At the terminal stage of prion infection miRNA-146a-5p, miRNA-142-3p, miRNA-143-3p, miRNA-145a-5p, miRNA-451a, miRNA-let-7b, miRNA-320, and miRNA-150-5p were elevated. During clinical stage of prion disease, all members of the miRNA-200 family (such as miRNA-200a-3p, miRNA-200b-3p, miRNA-200c-3p), miRNA-141-3p, miRNA-429-3p, and 182 cluster miRNAs (miRNA-182-5p and miRNA-183-5p) were downregulated (Boese et al., 2016). Similarly, an important inflammation related miRNA, miRNA-146a expression was upregulated in CJD and GSS patients (Lukiw et al., 2011). Bellingham et al. (2012) demonstrated that those exosomes, released by the prion-infected neuronal cells, had increased levels of let-7b, let-7i, miRNA-128a, miRNA-21, miRNA-222, miRNA-29b, miRNA-342-3p and miRNA-424 levels with decreased miRNA-146a levels compared to control exosomes in non-infected individuals.

Autophagy plays an essential role in cellular housekeeping processes via the degradation, recycling, and removal of old and damaged dysfunctional organelles and protein aggregates (Yoshimori, 2004). Impairments of autophagy/mitophagy, autophagy-lysosomal pathway and ubiquitin-proteasome system (UPS) has been studied extensively in prion diseases (Cortes et al., 2013; Choi et al., 2014; Mckinnon et al., 2016; Khan et al., 2017). Recently many research groups focused on the role of miRNAs in regulation of autophagy in several neurodegenerative diseases (Cheng et al., 2016; Shi et al., 2016; Li et al., 2017; Sun L. et al., 2017; Wang Y. et al., 2017; Yang et al., 2017). Kim et al. (2016) demonstrated that miRNA-27a and miRNA-27b plays a crucial role in the regulation of autophagy for clearing damaged mitochondria via PTEN-induced putative kinase 1 (PINK1) gene. Similarly, Cheng et al. (2016) demonstrated that miRNA-181a sensitizes neuroblastoma cells to apoptosis by suppressing Parkin-mediated mitophagy. miRNA-299-5p plays a crucial role in AD pathogenesis by regulating apoptosis through autophagy in N2a and SH-SY5Y cells and a mouse model of the AD (Zhang et al., 2016).

In this review article, we will overview our current understanding and knowledge about miRNAs-mediated regulatory roles in major neurodegenerative diseases. Furthermore, we will describe the role played by miRNAs in the regulation of autophagy in prion diseases and the therapeutic potentials of miRNAs in treating individuals afflicted with prion diseases.

## Biology of miRNAs

**FIGURE 1:**
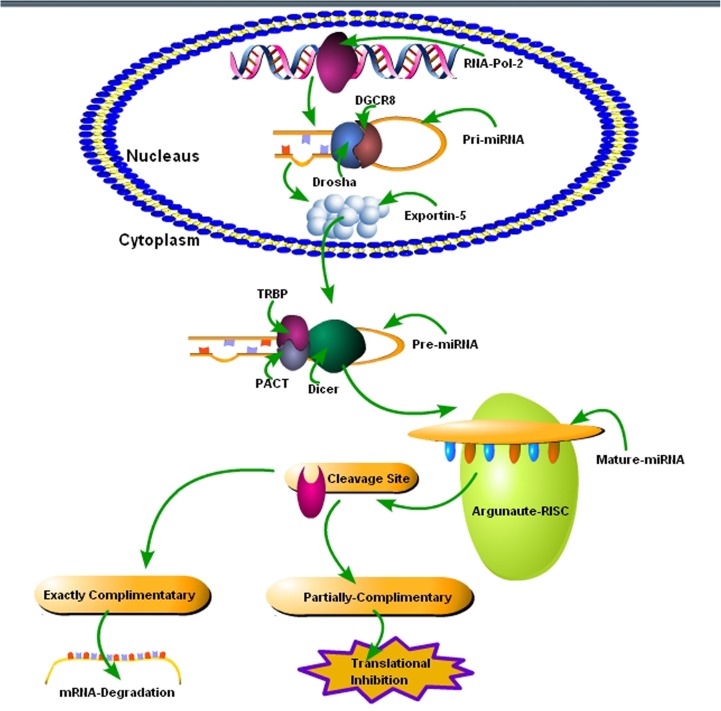
Schematic diagram of miRNA synthesis and biology. (1) In the first step, the transcription of pri-miRNAs occurs via *MIRNA* genes with the help of an RNA-polymerase-2 enzyme. (2) Pri-miRNAs size ranges from 70 to 100 nucleotides, and they fold into a hairpin-loop structure that consists of paired bases, which is composed of various mismatches and bulges. (3) The RNase III enzyme Drosha and its cofactor DGCR8/Pasha plays a crucial role in the excision of hairpin-loop structure to form pre-miRNAs. Ran-GTP-dependent exportin-5 protein helps in the transport of pre-miRNAs from the nucleus to the cytoplasm via nuclear pore complexes found in the nuclear membrane. (4) The pre-miRNAs is matured via RNase III, Dicer, in the cytoplasm. Dicer complex with transactivation responsive RNA binding protein (TRBP), protein activator of the interferon-induced protein kinase (PACT) cuts the pre-miRNAs and generates 21–23 nucleotide-long RNAs, which are mature miRNAs and ready to function. (5) Upon unwinding of the double helix, the mature miRNAs are joined to a ribonucleoprotein complex, known as “RNA-induced silencing complex” (RISC). The RISC complexes retain one of the strands as mature miRNA and eliminate the other strand. (6) Under the influence of Argonaute (AGO) family proteins, the mature miRNA targets the specific mRNA within the RISC. (7) Exact and partial complementation result in mRNA degradation and translation inhibition, respectively.

It was until the end of 20th century when the biological impacts of the small RNA molecules named as miRNAs by the research group of Prof. Victor Ambros opened up an entirely new avenue for molecular research (Lee et al., 1993). Since then, miRNAs are being considered and used as vital tools for the regulation of gene expression in many diseases (Lee et al., 1993). Molecular studies about their origin, biological synthesis, and activation mechanism have substantially advanced our knowledge about miRNAs. The biogenesis of miRNAs is a complex four-step process including transcription, nuclear processing, nuclear export and cytoplasmic processing. The transcription of miRNA genes usually occurs under the influence of RNA polymerase-2 enzyme found in the nucleus of eukaryotic cells. The RNA polymerase-2 enzyme binds with a promoter found near the DNA sequence; encoding would result in the hairpin loop of pre-miRNA (Lee et al., 2002; Reinhart et al., 2002; Gregory et al., 2004). The Animal miRNAs are initially transcribed as part of one arm of a ∼80 nucleotide RNA stem-loop that ultimately forms part of a several hundred nucleotide-long miRNA precursor known as primary miRNA (pri-miRNA). The presence of a stem-loop precursor in the 3′ UTR region will result in a transcript that may serve as pri-miRNA and a mRNA (Lee et al., 2002). There are one to six miRNA precursors in a single pri-miRNA. There are70 nucleotides each in these hairpin loop structures. Each hairpin is flanked by sequences necessary for efficient processing. The double-stranded RNA structure present in the hairpins of a pri-miRNA is recognizable by a nuclear protein called DiGeorge Syndrome Critical Region 8 (DGCR8 or “Pasha” in invertebrates). The DGCR8 associates with the enzyme Drosha, a protein that cuts RNA, to make a Microprocessor complex. The hairpins are liberated from pri-miRNAs by cleavage with RNA about 11 nucleotides from the base of the hairpin. The product that has 3′ hydroxyl and 5′ phosphate groups is termed as pre-miRNA. As much as 16% of the pre-miRNAs might be altered through nuclear RNA editing technique to alter downstream processing in the cytoplasm (Filipowicz et al., 2008; Huntzinger and Izaurralde, 2011). In next step, the pre-miRNA hairpins are exported from the nucleus in a process called nuclear export, which occurs under the influence of nucleocytoplasmic shuttler protein called exportin-5. The RNase-3 enzyme Drosha plays a vital role in the recognition of pre-miRNA hairpin via exportin-5. The transport to cytoplasm occurs under the influence of GTP bound to Ran protein. In the final step, the cytoplasmic processing is initiated, where the pre-miRNA hairpin is cleaved by RNase III enzyme Dicer. The Dicer complex interacts with the 5′ and 3′ end of the hairpin, and it cuts away the loop joining the 3′ and 5′ arms, making an imperfect miRNA:miRNA duplex of about 22 nucleotides in length. Although either of these strands may potentially act like a mature functional miRNA, only one of the strands is usually presented into the RNA-induced silencing complex (RISC), where the miRNA and its mRNA target interact (Lee et al., 2004). The RISC-bound miRNAs target its mRNA under the influence of the Argonaute (AGO) family proteins known as (Argonaute 1–Argonaute 4) (**Figure 1**) (Bartel, 2009). During the developmental stage of organisms, the specific cellular and tissues type have different levels of miRNAs. Apart from the endogenous stimulus, the exogenous environmental stimuli also play an important role in changing the levels of miRNAs (Aumiller and Forstemann, 2008). Abnormal miRNAs levels play a key role in the pathogenesis of neurodegenerative diseases. It might be possible that alterations in the biogenesis of miRNAs may provide important clues about the cellular and molecular mechanisms in these diseases; and it could be possible to generate novel targets based on miRNAs-mediated therapeutic interventions.

## miRNAs as Potential Biomarkers and their Credibility

The biomarker can be defined as a characteristic that is objectively measured and evaluated as an indicator of normal biological processes, pathogenic processes, or pharmacological responses to therapeutic intervention (Basak et al., 2016). The neurodegenerative disorders are highly heterogeneous in nature, and this heterogeneity necessitates the need for accurate diagnostic and prognostic biomarkers for these diseases. The molecular heterogeneity hampers the successful development of accurate and robust biomarkers for these diseases onsets and progressions. However, the recent findings that miRNAs are present in biofluids such as blood, cerebrospinal fluid, serum, and plasma (Gilad et al., 2008), as stable molecules has led to the development of recent studies based on miRNAs as potential candidates for biomarkers in several neurodegenerative diseases. To determine the potential of miRNAs as biomarkers for neurodegenerative diseases including prion diseases, we must examine the problems before implementing their routine use. A miRNAs should possess some of the basic characteristics of the ideal biomarker involving consideration of clinical, analytical, and practical criteria for implementation in these diseases. Some basic points or parameters before selection of miRNAs as biomarkers are:

(a) The optimal clinical sensitivity and specificity are important for selecting an ideal biomarker. The miRNA selected should provide high sensitivity in particular disease condition. In addition, an ideal miRNA expression profile must be demonstrated to be tissue-specific, however, this does not necessarily mean that translation is based on disease specificity as well.
(b) Detection and quantification of target miRNAs should be robust, rapid, simple, accurate, reproducible and inexpensive. Currently there are many issues regarding the use of miRNAs in neurodegenerative diseases as there is low correlation of results obtained from different platforms or even from the same platform using reagents from different companies. So the starting point should be standardization of these assays followed by normalization of data to get accurate results.
(c) The levels of biomarker should be proportional to the degree of severity of disease. This has been shown in various neurodegenerative diseases including AD, PD, and prion diseases, where the severity of disease has been shown to correlate with the levels of specific plasma or blood miRNAs.
(d) Minimal invasive procedures must be identified for obtaining the samples. The circulating miRNAs would merely require a blood sample, making it highly convenient for the patient, but in case of biopsy or as in all neurodegenerative diseases the brain tissue is affected, will make this process more difficult. The alternative could be obtaining postmortem brain tissues for sampling.
(e) The final and most important factor to remember is the pre-analytical variability of different samples. At present there are no guidelines for the collection, preparation, and extraction of samples for miRNA analysis. Different specimen types, such as brain tissue, cerebrospinal fluid, whole blood, plasma, or serum can introduce a variety of profound effects on miRNA concentrations. Biological variance is an important variable that may affect the clinical utility of miRNAs.

Before employing miRNAs as essential biomarkers for neurodegenerative diseases, we must understand the fact of species and tissue specificity. Roux et al. (2012) demonstrated that the expression of miRNAs was mostly tissue-dependent and the embryonic and nervous tissues shown little change in miRNAs expression with age. They further confirmed that the age of appearance was the primary factor affecting miRNAs expression (Roux et al., 2012). Rodent models are an important tool for molecular studies of neurodegenerative diseases. Human brain tissues and mouse models shown similarities in many aspects of transcriptome analysis but still, there are divergent points as well (Miller et al., 2010). Molecular studies related to miRNAs needs further molecular investigations in rodent models as most of the studies for miRNAs profiling were carried out on primate blood, serum, plasma, CSF or postmortem brain tissue from humans. In-depth studies on rodent models will surely show the extent of divergence amongst humans and rodents.

miRNAs are molecular biomarkers easily identifiable by using basic molecular identification techniques, such as genomics and proteomics. Genomics analysis includes northern blots for isolation of a set of RNA sequences. These RNA sequences can be analyzed by gene expression techniques or surveyed by using SAGE. The DNA microarray technology can be used to determine the frequency of each gene. The microarray and northern blots are usually consistent in data collection however at times; northern blots can detect small changes in gene expression that microarrays cannot detect (Taniguchi et al., 2001). On the other hand, microarrays are very popular for detecting a large number of genes as compared to northern blot (Taniguchi et al., 2001). Sample degradation by RNases from environmental contamination is a significant limitation in northern blot. Another limitation is the danger of chemicals used in northern blots such as formaldehyde, radioactive material, ethidium bromide, DEPC, and UV light are all harmful to the user. The polymerase chain reaction (PCR) technique is used to create many copies of the sequences for user-friendly approach. PCR is easy to use, a highly sensitive technique that can give results quickly but the major limitations are prior information about target sequence required, and DNA contamination will result in false positive results (Schochetman et al., 1988; Zhou et al., 1991). There are several proteomic techniques also available for identification of biomarkers such as 2D-PAGE, LC-MS, SELDI-TOF or MALDI-TOF, antibody array and tissue microarray. The tissue microarrays are combined with immunohistochemistry for cohort studies. Beside genomic and proteomic techniques there are other techniques used for the identification of biomarkers such as metabolomics, lipidomics, glycomics, and secretomics. Recently imaging techniques such as magnetic resonance imaging (MRI), computed tomography (CT), optical coherence tomography (OCT), positron emission tomography (PET) and near-infrared spectroscopy has also been used for the identification of biomarkers. MRI is generally safe technique, but scientists are advised not to overuse it, as it can cause injury due to foreign bodies in the patient’s tissue. MRI is preferred over CT, as it does not use any ionizing radiation. Although PET has gained the attention of many researchers working on AD and PD due to accurate glucose reactivity images, still there are specific limitations such as high costs of cyclotrons needed to produce short-lived radionuclides for PET.

It is evident from above discussion that not all biomarkers should be used as potential biomarkers for specific diseases and its outcome, and these biomarkers are difficult to validate. The biomarkers used for measuring the success of a therapeutic intervention must be directly related to the drug used.

## Regulation of miRNAs in Major Neurodegenerative Diseases

Neuroinflammatory and neurodegenerative events in major neurodegenerative diseases such as PD, AD, and prion diseases are complex processes related to a combination of changes on genetic, molecular and environment basis (Bossy-Wetzel et al., 2004). Neurodegeneration is still poorly understood in all protein misfolding diseases, but recent advances in neuroscience unraveled numerous pathways that play a crucial role in the pathogenesis of these diseases. With the advancement of latest molecular technologies and the involvement of miRNAs in stem cell differentiation, neuritogenesis, synaptoplasticity, etc. has broadened our understanding of the miRNAs biogenesis and using them from potential biomarkers to therapeutic intervention strategies are all underway (Kim J. et al., 2007; Manakov et al., 2009; Zhou et al., 2009; Gao et al., 2010; Bruno et al., 2011; Olde Loohuis et al., 2012) (**Table 1**).

**Table 1:** Showing a list of important biomarkers identified in Parkinson’s, Alzheimer’s, and prion Diseases (adapted from Basak et al., 2016 with minor modifications).

Disease	Source/number of +samples	Differentially expressed miRNAs	Method	Reference
Parkinson’s disease	Blood 32	miR-126-3p, miR-126-5p, miR-147, miR-151-5p, miR-151-3p, miR-199a-3p, miR-199a-5p, miR-19b, miR-26a, miR-28-5p, miR-29b, miR-29c, miR-301a, miR-30b, miR-30c, miR-335, miR-374a, miR-374b	Microarray, ChIP-seq	Martins et al., 2011
	Peripheral blood, plasma 15, 32, 42, 31	Blood-miR-1, miR-16-2-3p, miR-22-5p, mir-26a-2-3p, miR-29a, miR-30a Plasma-miR-1826, miR-450b-3p, miR-626, miR-505, miR-181c, miR-331-5p, miR-193a-p, miR-196b, miR-454, miR-125a-3p, miR-137	TaqMan low-density arrays, TaqMan assay, qRT-PCR, microarray	Khoo et al., 2011; Margis et al., 2011; Cardo et al., 2013
	Serum 25	miR-339-5p, miR-223-5p, miR-324-3p, miR-24, miR-30c, miR-148b	TaqMan low-density arrays, TaqMan assay	Vallelunga et al., 2014
	Serum 21	miR-338-3p, miR-30e-3p, miR-30a-3p, miR-16-2-3p, miR-1294	TruSeq small RNA sequencing	Lukiw, 2007
	White blood cells 8	miR-320a/b/c, miR-769, miR-92b, miR-16, miR-199b, miR-1274b, miR-21, miR-150, miR-671, miR-1249, miR-20a, miR-18b, miR-378c, miR-4293	Small RNA sequencing (ABI SOLiD)	Miller et al., 2013
	CSF 21	miR-132-5p, miR-19a-3p, miR-19b-3p, miR-127-3p, miR-409-3p, miR-370, miR431-3p, miR-873-3p, miR-136-3p, miR-10a-5p, miR-1224-5, miR-4448, let-7 g-3p, miR-128, miR-433, miR-485-5p, miR-212-3p	TruSeq small RNA sequencing,	Lukiw, 2007
	Brain 11	miR-34b, miR-34c	Microarray, qRT-PCR	Minones-Moyano et al., 2011
	Brain 3	miR-133b	qRT-PCR, northern blot analysis, luciferase assay	Kim J. et al., 2007
	Substantia nigra pars Compacta, 31	miR-198, miR-135b, miR-485-5p, miR-548d	TaqMan low-density arrays, TaqMan assay	Cardo et al., 2014
	Substantia nigra pars Compacta, 76	miR-26b, miR-106a, miR-301b, miR-21, miR-224, miR-373	qRT-PCR	Alvarez-Erviti et al., 2013
	Amygdala 43	miR-224, miR-373	qRT-PCR	Alvarez-Erviti et al., 2013
Alzheimer’s disease	Blood 32, 10	miR-34a, miR-181b	Microarray, qRT-PCR	Schipper et al., 2007
	Blood 7	miR-137, miR-181c, miR-9, miR-29a, miR29b,	qRT-PCR	Geekiyanage et al., 2012
	Peripheral Blood 215	miR-112, miR-161, let-7d-3p, miR-5010-3p, miR-26a-5p, miR-1285-5p, miR-151a-3p, miR-103a-3p, miR-107, miR-532-5p, miR-26b-5p, let-7f-5p	Next generation sequencing, qRT-PCR	Leidinger et al., 2013
	Plasma 31	let-7d-5p, let-7 g-5p, miR-15b-5p, miR-142-3p, miR-191-5p, miR-301a-3p, miR-545-3p	Nanostring, qRT-PCR	Kumar et al., 2013
	Serum 7	miR-137, miR-181c, miR-9, miR-29a, miR29b	qRT-PCR	Geekiyanage et al., 2012
	Serum 21	miR-125a-3p, miR-125b-1-3p, miR-127-3p, miR-1285, miR-135a-5p, miR-30c-2-3p, miR- 21-5p, miR-219-2-3p, miR-34c-5p, miR-34b-3p, miR-34b-5p, miR-22-5p, miR-375, miR-873, miR-1307-5p, miR-887, miR-182-5p, miR-184, miR-671, miR-3176	TruSeq small RNA sequencing,	Lukiw, 2007
	Brain, CSF 27, 20	miR-105, miR-10a, miR-10b, miR-143, miR-142-5p, miR-146b, miR-151, miR-125a, miR-126, miR-126, miR-127, miR-135a, miR-138, miR141, miR-181a, miR-181c, miR-15b, miR-154, miR-186, miR-191, miR-194, miR-195, miR-197, miR-199a, miR-204, miR-205, miR-214, miR-216, miR-221 ,miR-302b, miR-30a-3p, miR-30a-5p, miR-30b/c/d, miR-32, miR-99a, miR-501, miR-517a/b, miR-518b/f, miR-520a, miR-526a, miR-338, miR345, miR-362, miR-371, miR-374, miR-375, miR-380-3p, miR-422b, miR-429, miR-448, miR449, miR-451, miR-455, miR-494, miR-497, miR-7f	qRT-PCR	Cogswell et al., 2008
	CSF 6	miR-9, miR-125b, miR-146a, miR-155	Microarray	Alexandrov et al., 2012
	CSF 21	miR-10a-5p, miR-33b-5pmiR-101-5p, miR-124-3p, miR-127-3p, miR-127-5p, miR-132-3p, miR-129-5p, miR-134, miR-136-3p, miR-136-5p, miR-138-5p, miR-139-5p, miR-181a-5p, miR-181a-3p, miR-181b-5p, miR-181d, miR-184, miR-218-5p, miR-323a-3p, miR-326, miR-329, miR-377-5p, miR-381, miR-410, miR-431-3p, miR-433, miR-488-3p, miR-495, miR-708-5p, miR- 769-5p, miR-874, miR-9-3p, miR-9-5p, miR-95, miR-598, miR-760, miR-708-3p, miR-873-5p, miR-3200-3p	TruSeq small RNA sequencing,	Lukiw, 2007
	Brain 27	miR-125b, miR-106b, miR-107, miR-124, miR-132, miR-145, miR-146b, miR-148a, miR-17-5p	qRT-PCR	Cogswell et al., 2008
	Cortex 7	miR-137, miR-181c, miR-9, miR-29a, miR29b	qRT-PCR	Geekiyanage and Chan, 2011
	Cortex 10	miR-212, miR-424, miR-29a, miR-29b-1, miR-107, miR-15a	LNA-microarrays, northern blot analysis	Wang et al., 2011
	Cortex 34	miR-210, miR-320, miR-29a, miR-29b-1, miR-106b, miR-15a, miR-181c, miR-9, miR-22, miR-101, miR-197, miR-511, miR-19b, miR-26b, miR-363, miR-93, let-7i	Microarray, qRT-PCR, northern blot analysis	Hébert et al., 2008
	Cortex 21	miR-29a, miR29b, miR-338-3p	Microarray, qRT-PCR	Shioya et al., 2010
	Cortex 21	miR-101, miR-106b, miR-107, miR-125b, miR-137, miR-142-3p, miR-142-5p, miR-145, miR-151-5p, miR-15a, miR-181c, miR-184, miR-185, miR-194, miR-197, miR-19b, miR-210, miR-212, miR-214, miR-219-2-3p, miR-22, miR-223, miR-26b, miR-27b, miR-298, miR-29a, miR-29a/b-1, miR-29b-1, miR-300, miR-301a, miR-320, miR-326, miR-330-5p, miR-338-3p, miR-338-5p, miR-361-3p, miR-363, miR-382, miR	Microarray, qRT-PCR	Shioya et al., 2010
Prion Diseases	Brain 6	miR-342-3p, miR-320, let-7b, miR-328, miR-191, let-7d, miR-370, miR-128, miR-139-5p, miR-146a, miR-339-5p, miR-203, miR-181a-1^∗^, miR-338-3p, miR-337-3p, miR-200a, miR-200b, miR-26a, miR-186, miR-331-3p, miR-152, miR-221	Microarray, qRT-PCR	Saba et al., 2008
	Brain 6	miR-26a, miR-30a-5p, miR-30d, miR-103, miR-106b, miR-107, miR-124a, miR-125a, miR-128a, miR-132, miR-143, miR-145, miR-181a, miR-191, miR-195, miR-219, miR-320, miR-342-3p, miR-361, miR-490, miR-494	TaqMan low-density arrays, TaqMan assay	Montag et al., 2009
	Exosomes GT1-7 Cells	miR-126-3p, miR-134, miR-146a, miR-182, miR-186, miR-188-5p, miR-193b, miR-222, miR-296-3p, miR-29b, miR-380-5p, miR-424	qRT-PCR (TLDA cards)	Bellingham et al., 2012
	Exosomes GOTH-7 Cells	Let-7b, let-7i, miR-103, miR-125a-5p, miR-125b, miR-130a, miR-130b, miR-16, miR-21, miR-23a, miR-23b, miR-24, miR-296-6p, miR-29a, miR-29b, miR-29c, miR-301a, miR-30b, miR-30c, miR-342-3p, miR-344-4p, miR-378, miR-93	RNA sequencing	Bellingham et al., 2012

Parkinson’s disease equally affects both younger, and older human populations across the world and an estimated 50,000 Americans are diagnosed each year with PD. PD is a chronic motor neurodegenerative disease with incidence rate almost as high as AD. There are several genes responsible for inherited PD, but five widely accepted genes responsible for late-onset are a-synuclein (SNCA), and leucine-rich repeat kinase 2 (LRRK2), whereas, for early onset, Parkin (PARK2), oncogene DJ1, and PTEN-induced PINK1 are important (Coppede, 2012). LRRK2 is downregulated by miR-205, and pathogenic mutations in the LRRK2 disrupts the critical signaling mechanism of let-7 and miRNA-184, which causes the deregulation of transcription factor E2F1/DP and cell survival is hampered (Gehrke et al., 2010; Cho et al., 2014). Minones-Moyano et al. (2011) demonstrated that miRNA-34b/c regulate DJ-1/PARK7 and Parkin protein expression in SHSY-5Y cell model and in the brains of patients afflicted with PD. Several research groups demonstrated that miRNA-548d, miRNA-224, miRNA-373, miRNA-198, miRNA-106a, miRNA-26b, and miRNA-301b show altered expression in PD patients (Alvarez-Erviti et al., 2013; Burgos et al., 2014; Cardo et al., 2014). The midbrain samples showed miR-133b downregulated in PD patients. Dopaminergic neurons are enriched with Pixt3, which is a crucial transcription factor and miRNA-33b directly increases the expression of Pixt3 (Goodall et al., 2013). Alpha-synuclein (SNCA) plays a vital role in the pathogenesis of PD. In addition, miR-7 and miR-153 target the 3.0 UTR of SNCA, bind directly to SNCA mRNA to downregulate its expression and are enriched in the brains of PD patients (Doxakis, 2010). A polymorphism in the expression of miRNA-433 increases the Fibroblast Growth Factor 20 (FGF20) gene expression. FGF20 ultimately upregulates the downstream target SNCA (Itoh and Ohta, 2013). Li et al. (2014) demonstrated that miRNA-320a plays an essential role in SNCA aggregation during PD pathogenesis.

Alzheimer’s disease is one the primary neurodegenerative disease affecting 44 million people around the world, and statistics reveal that only one-in-four people affected with AD have been diagnosed (Basak et al., 2016). Several studies demonstrated that miRNA-148a, miRNA-17-5p, miRNA-137, miRNA-181c, miRNA-101, miRNA-184, miRNA-15a, miRNA-185, and miRNA-210 are few of those miRNAs that are expressed in AD (Lukiw, 2007; Cogswell et al., 2008; Hébert et al., 2008; Geekiyanage and Chan, 2011; Wang et al., 2011). Tauopathies involved in the pathogenesis of AD have many key proteins, which are regulated by miRNAs. Dickson et al. (2013) demonstrated that endogenous tau aggregates were repressed by miRNA-34a in M17D cell model of the AD. Similarly, many researchers found that the amyloid precursor protein (APP) expression is also influenced by miRNA-101, miRNA-16, miRNA-106a, and miRNA-644 (Patel et al., 2008; Delay et al., 2011; Long and Lahiri, 2011; Liu et al., 2012). Furthermore, the amyloid protein fragment amyloid beta (Aβ), is also influenced by miRNA-24, miRNA-186, miRNA-455, miRNA-146a, and miRNA-98 (Delay et al., 2011; Li Y.Y. et al., 2011; Hu et al., 2013). The levels of Aβ in AD patients have been speculated to be regulated by miRNA-137, miRNA-146a, and miRNA-181c (Delay et al., 2011; Geekiyanage and Chan, 2011; Li Y.Y. et al., 2011; Hu et al., 2013). Interestingly, there are many miRNAs, such as miRNA-107, miRNA-29a-1/b-1, miRNa-9 and miRNA-124, which regulate the APP-processing enzyme b-secretase enzyme I (BACE1) in AD patients (Bossy-Wetzel et al., 2004; Wang et al., 2010; Fang et al., 2012). Some miRNAs have a substantial influence on age-related disorders such as AD. The anti-aging factor sirtuin-1 (SIRT1) plays a vital role in enhancing the neuronal survival and alleviating neurodegeneration in AD and ALS models. The SIRT1 expression is also regulated by miRNA-34a (Kim D. et al., 2007; Li X. et al., 2011; Smit-McBride et al., 2014). The demented aging brain showed an inverse relationship between SIRT1 and miRNA-34a. Similarly, it has been demonstrated that miRNA-144 can possibly bind to the 3UTR of programmed cell death protein 4 (PDCD4), which shows that miRNA-144 might be crucial in apoptotic mechanisms during AD development (Persengiev et al., 2011). Recently Wang Y. et al. (2017) showed that miRNA-132/212 impaired *S*-nitrosylation and induced tau phosphorylation via NOS1 pathway. Similarly, Lau et al. (2013) studied 90 samples of human brain affected with the AD, and found that miRNA-132-3p was strongly downregulated. In addition, the transcription factor FOXO1a was the target of miRNA-132-3p, which plays a crucial role in tau network (Lau et al., 2013). Song and Kim (2017) showed that miRNA-142-5p plays an important role in the pathogenesis of AD, as it is upregulated in the brains of AD patients and inhibition of miRNA-142-5p resulted in the rescue of synaptic dysfunction in Aβ42-treated SH-SY5Y cells. Zhang et al. (2017) demonstrated that miRNA-200a-3p promotes Aβ-induced neuronal apoptosis via downregulation of SIRT1 in a transgenic mouse model of the AD and PC12 cells. Kumar et al. (2017) demonstrated that upregulation of miRNA-455-3p, miRNA-4668-5p, miRNA-3613-3p, and miRNA-4674, while downregulation of miRNA-6722 in 10 AD positive brain samples.

## Regulation of miRNAs in Prion Diseases

While the misfolded protein diseases often feature complex interactions between aggregates of multiple proteins, all misfolded proteins share a common structural feature, known as the amyloid folds (Andreoletti et al., 2000, 2002). Prion disease pathogenesis is complex, and the involvement of miRNAs deregulation in prion disease pathogenesis is still unclear. It is demonstrated that during preclinical stage of prion disease, miRNAs abundant in synaptoneurosomes including miRNA-124a-3p, miRNA-136-5p and miRNA-376a-3p are increased. While in terminal stage of prion infection miRNA-146a-5p, miRNA-142-3p, miRNA-143-3p, miRNA-145a-5p, miRNA-451a, miRNA-let-7b, miRNA-320, and miRNA-150-5p are significantly elevated in the brains of prion-infected animals. miRNA-124 and miRNA-126 are also upregulated in prion, AD and PD patients (Lukiw, 2007; Cogswell et al., 2008; Martins et al., 2011; Bellingham et al., 2012). During clinical phase of prion disease all the members of the miRNA-200 family (miRNA-200a-3p, miRNA-200b-3p, and miRNA-200c-3p), miRNA-141-3p, and miRNA-429-3p and the 182 cluster (miRNA-182-5p and miRNA-183-5p) are downregulated (Boese et al., 2016). Similarly, miRNA-146a expression is upregulated in CJD and GSS patients (Lukiw et al., 2011). Recently, miRNA-200 has been found upregulated in AD models (Zhang et al., 2017). Bellingham et al. (2012) demonstrated that let-7b, let-7i, miRNA-128a, miRNA-21, miRNA-222, miRNA-29b, miRNA-342-3p, and miRNA-424 levels were upregulated, while miRNA-146a levels were downregulated in prion-infected individual. Different miRNAs profiles were observed in the brains of BSE- infected cynomolgus macaques (Montag et al., 2009). Through miRNA-microarray and qRT-PCR analysis, two miRNAs miRNA-342-3p and miRNA-494 were significantly upregulated in the brains of BSE-infected macaques compared to non-infected animals. In addition, the miRNA miRNA-342-3p was also upregulated in the brains of human type 1 and type 2 sporadic CJD patients. Similarly, miRNA-342-3p was also upregulated in scrapie-infected mice brains (Saba et al., 2008; Montag et al., 2009). These studies reveal that miRNA-342-3p might be a potential biomarker in the terminal stage of prion diseases. No validation of this hypothesis exists, due to the lack of knowledge about the target mRNAs for miRNA-342-3p. Although, the TargetScan algorithm shows some important genes targeted by miRNA-342-3p, such as ataxin 7, tau tubulin kinase 2 and huntingtin interacting protein 1 (HIP1). It is worthwhile to note, that these genes are primarily involved in the protein misfolding disorders. Further confirmation of the significance of miRNA-342-3p in prion diseases is required due to the small number of experimental animals and human brains available for the above-mentioned study (two sCJD and one healthy control) (Saba et al., 2008). Similarly, miR-342-3p and miR-21-5p are upregulated in the brains of sheep naturally infected with prion disease (Sanz Rubio et al., 2017).

Neuroinflammation and neurodegeneration are the hallmarks of prion diseases, and miRNA-146a plays a crucial role in activating the innate immune response and microglial activation during the course of prion diseases (Saba et al., 2012). Saba et al. (2012) showed that miRNA-146a is overexpressed during prion infection and microglial lineage cell lines, such as BV2 are directly modulated via TLR2 or TLR4 receptors. miRNA-146a mimic decreased the expression of interleukin-1 beta (IL-1β) and interleukin-18 (IL-18), while increased the expression of interleukin-10 (IL-10) (Saba et al., 2012). The role of miRNA-146a in the regulation of inflammatory responses during prion diseases and its modulatory effect on nuclear factor-kappa and the Janus kinase (JAK) signal transducer and activator of transcription (JAK-STAT) signaling makes it a new and attractive therapeutic target for prion diseases (**Figure 3**) (Saba et al., 2012). Similarly, overexpressed miRNA-146 is observed in prion and AD patients (Cogswell et al., 2008; Bellingham et al., 2012). During the preclinical phase of prion disease, a cluster of genes and miRNAs are dysregulated, such as miRNA-132-3p, miRNA-124a-3p, miRNA-16-5p, miRNA-26a-5p, miRNa-29a-3p, and miRNA-140-5p, and they follow associated patterns of expression (Majer et al., 2012). miRNA-125 is upregulated in prion, AD, and PD models (Cogswell et al., 2008; Bellingham et al., 2012; Cardo et al., 2013). Recently, Gao et al. (2017) worked on miRNA expression profile of three prion strains, 139A, ME7, and S15. They found that the most significantly upregulated and downregulated miRNAs in scrapie-infected mice were, mmu-miRNA-3473e (9.48 log_2_) and mmu-miRNA-141-5p (-7.33 log_2_) in the 139A group, mmu-miRNA-3473e (13.18 log_2_) and mmu-miRNA-200a-5p (-7.08 log_2_) in the ME7 group, and mmu miRNA-3473e (14.15 log_2_) and mmu-miRNA-183-3p (-10.15 log_2_) in the S15 group (Gao et al., 2017). Recently, Burak et al. (2018) demonstrated that miRNA-16 localized within the CA1 region of the hippocampus was upregulated in the early stage of prion infection. The novel targets of miRNA-16 were APP, BCL2, MAPK, and ERK. They showed that miRNA-16 plays a crucial role in neurite growth and branching (Burak et al., 2018). Similarly, Miller et al. (2013) also showed the upregulation of miRNA-16 in PD patients. Llorens et al. (2018) demonstrated the miRNA signature in frontal cortex (FC) and cerebellum (CB) of sporadic CJD patients. In sCJD FC, miRNAs 29b-3p, 342-3p, 146a-5p, 154-5p, 195-5p, 26a-5p, 16-5p, 449a, 142-3p, let7i-5p, and 135a-5p were upregulated, while miRNAs 124-3p, 331-3p, 877-5p, and 125a-5p were downregulated compared to controls. miRNAs 378a-3p and 5701, which expression was only altered in CB did not present changes in the FC. In CB, miRNAs 146a-5p, 154-5p, 26a-5p, 378a-3p, 449a, 142-3p, let7i-3p, and 5701 were upregulated, and miRNAs 124-3p and 877-5p were downregulated in sCJD. The rest of miRNAs, which expression was only altered in FC did not present changes in the CB (Llorens et al., 2018).

## Degradation Pathways for Aggregated Prion Proteins

The essential cellular quality control pathways for prion protein degradation are ubiquitin-proteasomal system (UPS) and autophagy-lysosomal pathways (Goold et al., 2015; Shah et al., 2017b). UPS involves the attachment of ubiquitin chains, which are then recognized by proteasome for subsequent degradation. The substrate proteins are ubiquitinated in a three-step mechanism: firstly, an enzyme (E1) which activates the ubiquitin covalently binds with ubiquitin moiety in an ATP-dependent manner, secondly; already formed ubiquitin moiety is then exposed to another enzyme called E2 ubiquitin conjugation enzyme. In the third and final step, a third enzyme called E3 ubiquitin ligase controls the reaction based on the specificity of the substrate. These ubiquitination steps are run in several rounds to prolong the ubiquitin chain, and it will result in polyubiquitination of the target substrate protein. There are various modes of ubiquitination, dependent on the lysine residue that is used for connecting with different ubiquitin moieties. The proteasomal degradations are carried out via lysine-48 (K48). The substrates recognized for ubiquitination through K48 are targeted by the proteasome ubiquitination system, and they are eventually unfolded and degraded. While the ubiquitin used for all this process is cleaved off and recycled (Kang et al., 2004; Goold et al., 2015).

**FIGURE 2:**
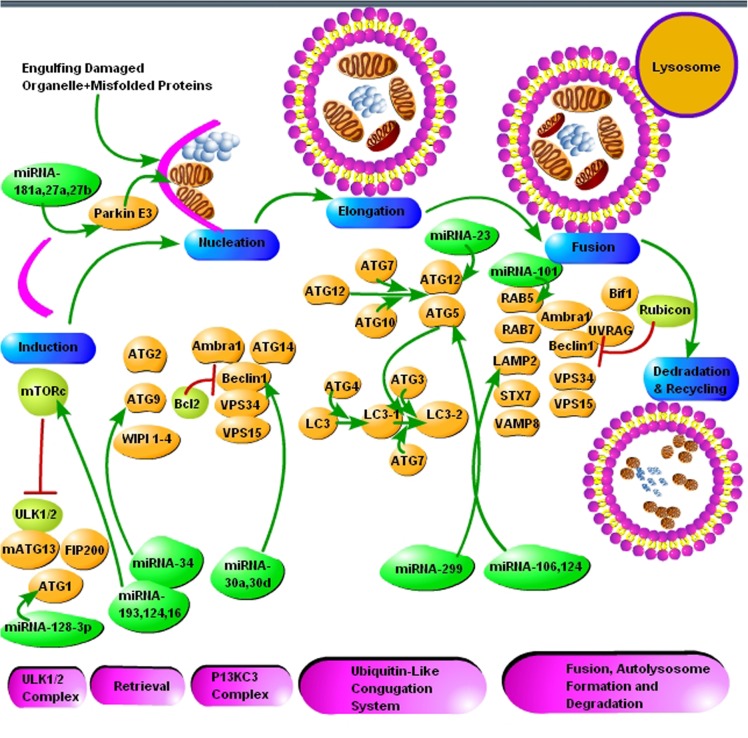
The schematic presentation of autophagy pathway and core autophagy-related proteins targeted by various miRNAs. The figure shows some significant miRNAs (dark green color) and their respective targets (light green and yellow color) for the regulation of autophagic mechanisms in Alzheimer’s and Parkinson’s disease. The figure shows that insufficient research has been conducted in neurodegenerative diseases for the regulation of autophagy via miRNAs and further molecular research is encouraged in all major neurodegenerative diseases including prion diseases.

Autophagy is a bulk degradation process that starts with the development of a crescent shape double membrane known as phagophore, which then engulfs the damaged organelle or cargo, which is supposed to be degraded to form autophagosome. The autophagosome gets mature and fused with a lysosome to form autolysosome. The acidic lysosomal proteases degrade the cargo within the autolysosome (**Figure 2**) (Goold et al., 2015). Ubiquitin molecules also tag the substrate recognized for degradation by autophagy. Substrates are labeled by the ubiquitin and like UPS, which is dependent on the lysine residue; here the linkage is via lysine-63 (K63), and ultimately the autophagy initiation signals are started. The autophagy adaptor proteins recognize the polyubiquitin signals which mediate and simultaneous bind the ubiquitinated cargo with the autophagic machinery. Various adaptor proteins were recognized in different species including yeast and in mammalian cells. All these adapter proteins are characterized by the ubiquitin-binding domain. The best example of which is SQSTM1/p62 protein. The p62 protein posses a domain called ubiquitin-associated domain (UBA). UBA is involved in ubiquitin binding and an LC3-interacting region (LIR), which attaches with autophagosomes. Several researchers demonstrated that p62 protein plays a crucial role in the degradation of misfolded aggregated proteins and mitophagy (Ding et al., 2010; Geisler et al., 2010; Tung et al., 2010). Many scientists used p62 as a marker for staining the protein aggregates in various proteinopathies, including neurodegenerative diseases. This further elaborates the role of selective autophagy in the pathogenesis of misfolded protein aggregation diseases (Zatloukal et al., 2002) Neurodegenerative diseases like prion, AD, PD and Huntington’s disease are characterized by the deposition of intra and extracellular protein aggregation, which cannot be degraded by proteasomes. Thus, activation of autophagy is seen as an alternative therapeutic strategy for protein aggregation diseases.

**FIGURE 3:**
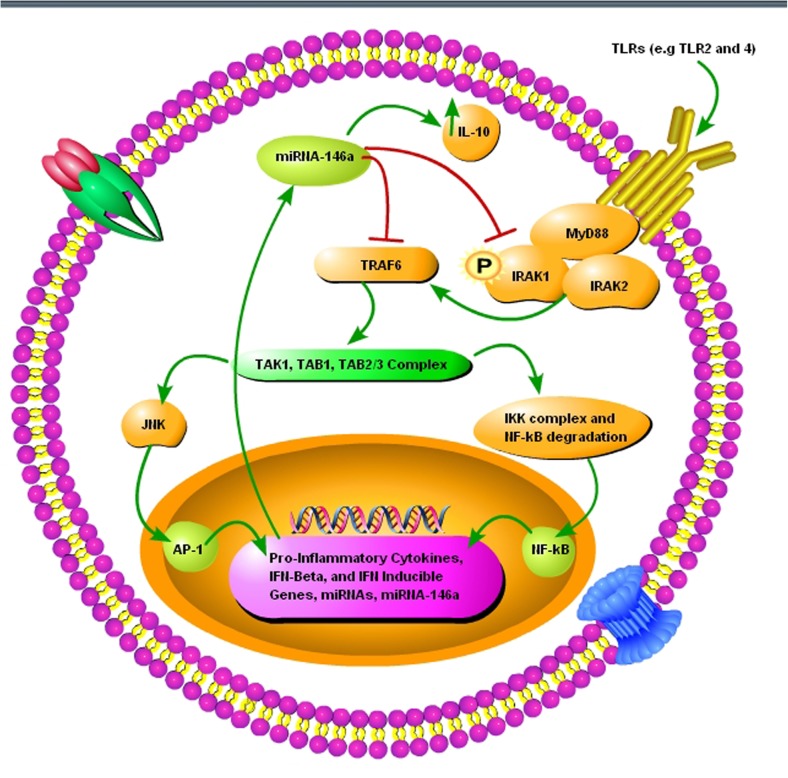
A schematic diagram showing the importance of pro-inflammatory miRNA-146a during prion infection. (1) The toll-like receptors TRL2 and TLR4 are activated upon misfolded prion protein formation. (2) TNF receptor-associated factor 6 (TRAF6) and interleukin-1 receptor-associated kinase 1 (IRAK1), interleukin-1 receptor-associated kinase 2 (IRAK2), myeloid differentiation primary response gene 88 (MyD88) complexes are modulated in response to activated TLR2 and TLR4 receptors. (3) TRAF6 activates the c-Jun N-terminal kinases (JNK) and IκB kinase (IKK) complex via TGF-beta activated kinase 1 (TAK1), TAK1-binding protein 1 (TAB1), and (TAB2/3) complex. (4) NF-κB degradation and nuclear translocation plus activator protein 1 (AP-1) activation via JNK result in upregulation of proinflammatory cytokines and several miRNAs including miRNA-146a. (5) Upregulated miRNA-146a inhibits IRAK1 and TRAF6 signaling to increase interleukin-10 (IL-10) secretion and reduce inflammation.

## Role of Autophagy in Prion Diseases

Autophagy is a primary cellular housekeeping process for the degradation and recycling of cellular components that maintains energy homeostasis via degradation of aggregated proteins within the cell (Clarke, 1990; Yoshimori, 2004). The induction of autophagic flux plays an essential role in the degradation of misfolded prion proteins (Yao et al., 2013). Any cellular modification leading to the disruption in autophagic mechanisms via the use of pharmacological interference or siRNA gene silencing dramatically reduce the efficiency of compound-induced autophagy, and ultimately the cellular levels of PrP^Sc^ remained high (Heiseke et al., 2010). Recent molecular techniques such as small molecules for the induction of autophagy have also shown the protective mechanism of autophagy in neurodegenerative diseases. Heiseke et al. (2009) demonstrated that lithium-induced clearance of PrP^Sc^ within the prion-infected cells was solely dependent on increased autophagy via mTOR-independent manner (Yao et al., 2013). Recently Moon et al. (2016b) demonstrated that plant extract hinokitiol successfully alleviated the prion-peptide induced toxicity via restoration of autophagic flux in primary neurons. Similarly, ginsenoside-rg3 attenuated prion peptide-induced neurotoxicity via autophagic flux through modulation of mitochondrial dysfunction (Moon et al., 2016a).

Mice treated with autophagy inducer rapamycin showed a prolonged incubation period in comparison with the vehicle-treated control mice (Cortes et al., 2012). Similarly, autophagy was protective against *de novo* formation of PrP^Sc^ in a yeast model of prion diseases, and increased autophagic flux resulted in the degradation of aggregated PrP^Sc^ proteins (Speldewinde et al., 2015). In another study, rapamycin reduced the quantity of misfolded PrP^Sc^ in a mTOR-dependent manner. Rapamycin treatment significantly improved mitochondrial dysfunction, neuroinflammation, and neurodegeneration in a mouse (Johnson et al., 2013). Recently Wang H. et al. (2017) demonstrated that overexpression of Polo-like kinase 3 (PLK3) played an important role in the degradation of PrP^Sc^ proteins. Some researchers proposed that beneficial effects of rapamycin are related to the indirect inhibition of protein synthesis, ultimately maintaining the ATP levels within the infected cells (Zheng et al., 2016). Modern molecular techniques such as small molecules can stimulate autophagy in mTOR-dependent and mTOR-independent manners. This shows the importance of small molecules for the possible treatment of patients with prion diseases by targeting mTOR-dependent and mTOR-independent pathways simultaneously. More detailed *in vivo* studies showing the prospects of small molecules as a treatment for prion diseases are necessary.

The miRNAs contribute to the development of neurons, neuritogenesis and synapse growth. miRNAs are also involved in the CNS ischemia, spinal cord injury, and traumatic brain injury (Bellingham et al., 2012). The prion gene-associated miRNAs play a vital role in neuronal apoptosis, differentiation, development, synaptic integrity, and neurogenesis (Shapshak, 2013). Studies involving prion-infected mouse brain showed that various gene promoters are related to miRNAs. Some important gene promoters identified were E2F-1 (cell cycle re-entry and neurodegeneration), MAZ (inflammatory response transcription factor), PAX6 (neurogenesis), KROX (transcription factor), and early growth response 1 (EGR1). Additionally, human studies involving the brains of individuals affected with prion diseases showed the effect on miRNAs on *N*-methyl-D-aspartic acid (NMDA) receptors and glutamate receptors (ionotropic, *N*-methyl-D-aspartate 2A, GRIN2A) (Majer et al., 2012; Saba et al., 2012).

Perturbation in the clearance of misfolded proteins by autophagic flux and UPS is the hallmark of all prion disorders. The UPS pathway is comprised of over 30 genes that are involved in the dysregulated miRNAs and been identified in prion diseases (Saba et al., 2008). Some of these important genes are involved in ubiquitin, ubiquitin-conjugating enzymes, and the ubiquitin protein ligase NEDD4. Unfolded-protein response genes are also the primary targets, such as valosin-containing protein, VCP (CDC48/p97). VCP plays an essential role in the shuttling of ubiquitinated proteins from the endoplasmic reticulum (ER) to the proteasome system, and the transcription factor X-box binding protein 1, XBP1, activated upon the accumulation of misfolded proteins in the ER (Saba et al., 2008).

Neuroinflammation is the hallmark of all protein misfolding diseases including prion diseases, and one genuinely noteworthy finding is that a considerable upregulation of inducible, pro-inflammatory pathogenic miRNAs such as miRNA-34a and/or miRNA-146a are collectively shared by AD and prion diseases (Hill and Lukiw, 2015; Zhao et al., 2015a,b). As these pro-inflammatory miRNAs are also crucial for plaque formation during AD and prion diseases, it could be involved in the regulation of autophagic mechanism during prion diseases. The detection of those miRNAs involved in the pathogenesis of disease process and the following interacting proteins provide several important junctures that might be targeted in devising treatment strategies and possible cures for prion diseases.

## Role of miRnas in Modulating Autophagy: Enlightenment for Prion Diseases

Protein aggregation or accumulation is a biological phenomenon commonly observed in neurodegenerative disorders known as amyloidosis, including ALS, AD, PD, and prion disease. The clearance of these aggregated proteins is necessary for cell survival and functioning. The aggregated proteins are mainly cleared through the UPS (Yedidia et al., 2001), but severe ER stress and a significant amount of aggregated proteins lead to dysfunction of the UPS (Jellinger, 2010). The failure of the UPS leads to triggering another clearance mechanism known as autophagy (**Figure 2**) (Lynch-Day et al., 2012). Defective UPS and autophagy are linked to the pathogenesis of AD, PD, and prion diseases (Kristiansen et al., 2007; Schapira and Gegg, 2011).

Autophagy does not degrade miRNAs directly, but it influences the proteins targeted by miRNAs and hence miRNAs-mediated repression of appropriate target genes is hampered. Gibbings et al. (2012) demonstrated that autophagy selectively degrades miRNA-processing enzyme, Dicer1 and the main miRNA effector protein, Ago2, by selective autophagy receptor NDP52, ultimately regulating miRNAs biogenesis. This shows that NDP52 and autophagy plays a crucial role in the homeostasis and activity of miRNAs (Gibbings et al., 2012). Similarly, it is demonstrated that miR-106a and miR-224 are upregulated in the brains of PD patients, ultimately leading to impaired chaperone-mediated autophagy (CMA) and α-synuclein accumulation. miR-106a and miR-224 cause a dose-dependent reduction in heat shock 70 kDa protein (hsc70) and lysosome-associated membrane protein 2 (LAMP-2A), respectively, to impair autophagic mechanism in SH-SY5Y cells (Alvarez-Erviti et al., 2013). Both hsc70 and LAMP-2A are active mediators of autophagy, and they play a crucial role in CMA (Majeski and Dice, 2004). Li et al. (2014) showed that miRNA-320a targeted the 3′ UTR of hsc70, decreased hsc70 expression in α-synuclein-overexpressed SH-SY5Y cells, and resulted in significant α-synuclein intracellular accumulation. miRNA-34 mutations extend the life of *Caenorhabditis elegans* by increasing autophagic flux. Furthermore, miR-34 represses autophagy by directly inhibiting the expression of the autophagy-related protein Atg9 in mammalian cells (Yang et al., 2013). Recently, Valera et al. (2017) demonstrated that overexpression of miRNA-101 in oligodendroglial cell cultures resulted in a significant increase of α-synuclein via disruption of autophagy. The predicted target of miRNA-101 was autophagy-related gene RAB5A. The stereotaxic injection with anti-miRNA-101 into the striatum of a mouse model of multiple system atrophy resulted in reduced oligodendroglial α-syn accumulation and enhanced autophagy (Valera et al., 2017). The role played by miRNAs in the regulation of autophagy is further supported by the work where essential autophagy-promoting protein Beclin 1(BECN1) was directly regulated by miRNA-30a. Furthermore, miRNA-206 regulated HDAC4, which is linked with autophagy (Williams et al., 2009; Zhu et al., 2009). Another miRNA known as miRNA-29a/b-1, downregulated in the brains of sporadic AD patients causes dysregulation of proteins like, APP or BACE1/β-secretase, resulting in an autophagy-dependent cell toxicity and death events (Hébert et al., 2008).

Li et al. (2017) demonstrated that miRNA-193b-3p plays a vital role in the pathogenesis of ALS. Downregulation of miRNA-193b-3p promoted autophagy and cell survival by targeting TSC1/mTOR signaling pathway in NSC-34 cell model of ALS. In contrast, upregulation of miRNA-193b-3p activated mTORC1 signaling leading to the inhibition of autophagy and promoted cell death (Li et al., 2017). Antagomirs of miRNA-134 rescued epileptic rats from lithium chloride-pilocarpine-induced status epilepticus via regulation of oxidative stress, mitochondrial dysfunction, and autophagy. Anti-134 treatment downregulated the expression of autophagy-associated proteins ATG 5, Beclin-1, and LC3-b, to enhance memory and survival in mouse model of epilepsy (Sun J. et al., 2017). miRNA-30d rescued rat astrocytes from oxygen and glucose deprivation by targeting Beclin 1 in cell injury under hypoxic condition (Zhao F. et al., 2017). miRNA-124 participates in neuronal protection against ischemic stroke by targeting P13K/Akt signaling pathway in PC12 cells (Wang C.M. et al., 2017; Wang Y. et al., 2017). Moreover, miRNA-124 is protective of dopaminergic neurons via regulation of AMPK/mTOR pathway in SH-SY5Y cell model of PD (Gong et al., 2016). Sun L. et al. (2017) showed that miRNA-23b improved cognitive impairment in a traumatic brain injury rat model by targeting ATG 12-mediated neuronal autophagy. miRNA-299-5p downregulation is observed in APPswe/PS1dE9 mice, cerebrospinal fluid of AD patients, N2a and SH-SY5Y cell models of the AD. It is demonstrated that miRNA-299-5p regulates autophagy by targeting ATG 5 directly (Zhang et al., 2016). The role of miRNA-16 in reversing autophagic and apoptotic changes during chronic stress are studied recently in a rat model of chronic stress. It is observed that miRNA-16 restores BDNF levels by downregulating p13-Akt-mTOR pathway (Yang et al., 2017). The involvement of miRNA-128-3p expression by directly targeting ATG 1 in cerebral ischemia rat model has been established recently (Shi et al., 2016). Cheng et al. (2016) found that miRNA-181a is a novel inhibitor of mitophagy and it directly targets Parkin E3 ubiquitin ligase. Similarly, miRNA-181a regulated apoptosis and autophagy in PD by inhibiting the two crucial p38 mitogen-activated protein kinase (MAPK)/c-Jun N-terminal kinases (JNK) signaling pathways (Liu et al., 2017). miRNA-27a and miRNA-27b play a vital role in the autophagic clearance of injured mitochondria by targeting PTEN-induced PINK1 in PD (Kim et al., 2016).

Kang et al. (2017) demonstrated that dual miRNA to cellular prion protein successfully inhibited the propagation of pathogenic prion in a mixed culture of neuronal and glial cells. Furthermore, this reduction in PrP^Sc^ was cell type-specific as no reduction was observed in reserve cells. Interestingly, a 20% decrease in PrP^c^ levels led to more than 60% decreases in pathogenic misfolded form PrP^Sc^ (Kang et al., 2017). Although, this study did not focus on the role played by autophagy in the degradation of misfolded prions. However, it encourages the researchers that if an artificial dual miRNA and siRNA combination can reduce the pathogenic PrP^Sc^ levels substantially, then there might be prospects for finding the naturally present miRNAs that have an important role in targeting the autophagy-related proteins, and ultimate goal of PrP^Sc^ free cell can be achieved.

Our research group focused on the activity of repressor element-1 binding transcription factor (REST), or neuron-restrictive silencer factor (NRSF), in prion diseases (Song et al., 2016, 2017a,b). REST is an important monitor for the establishment of neuronal specificity, and it is highly expressed in neuronal tissues (Zhao Y. et al., 2017). Interestingly, REST is the target of many brain-derived miRNAs such as miR-124a, miR-9, and miR-132 (Wu and Xie, 2006). Lu et al. (2014) demonstrated that REST expression in the nucleus of neurons in the AD, frontotemporal dementia, and dementia with Lewy bodies patients was lower as compared to age-matched controls. REST is misplaced from the nucleus and can be found in autophagosomes together with pathological misfolded proteins (Lu et al., 2014). We found similar results with the PrP peptide 106–126-induced neurotoxicity in primary neurons and N2a cells after 24 h exposure (Song et al., 2016). Cho et al. (2014) demonstrated that REST regulates mTOR-signaling pathway in oral carcinoma. We also found REST mediated regulation of Akt-mTOR and Wnt-β-catenin pathway in 263K infected hamsters (Song et al., 2017a). We can speculate that REST-directed miRNA therapies in neurodegenerative diseases may be practical in regulating autophagic flux. The knowledge from these recent studies could play a crucial role in finding the therapeutic remedy toward autophagy regulation in prion-related neuropathologies as well.

## Modern Molecular Techniques to Target miRnas for Therapeutic Purpose

**FIGURE 4:**
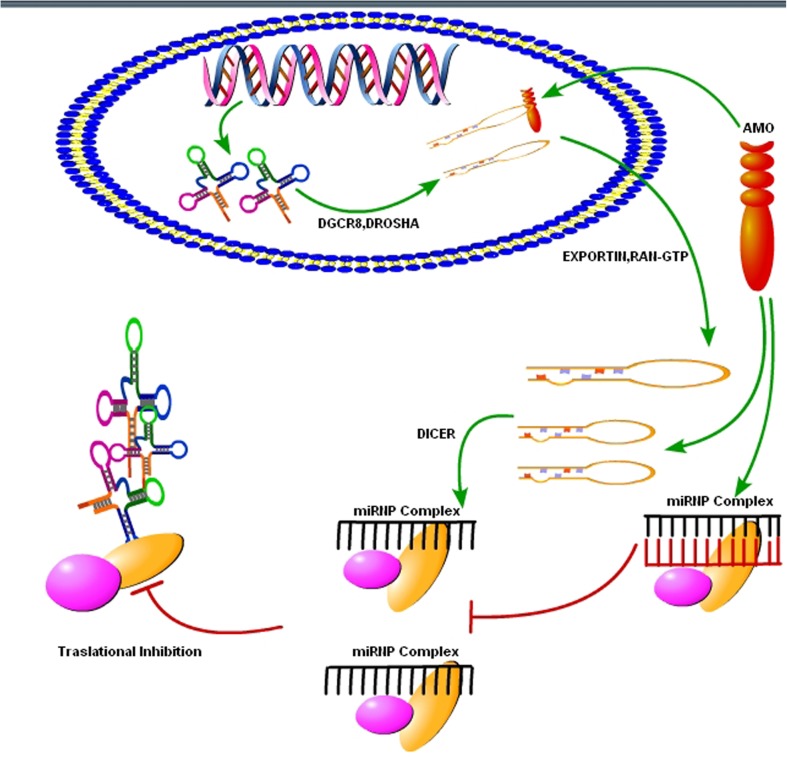
Use of synthetic oligonucleotides to interfere the activity of miRNAs. The Inhibition of the activity of miRNA may be achieved by using anti-miRNA oligonucleotides (AMOs) fully complementary to the pri-miRNA, the pre-miRNA or the mature miRNA (figure adapted from Weiler et al., 2006).

As we know that upregulation or downregulation of a miRNA is dependent on the expression of a specific gene, so modern molecular techniques such as Clustered, Regularly Interspaced, Short Palindromic Repeats (CRISPER) -CRISPR-associated 9 (Cas9) can be used to control the expression levels of specific miRNAs. The CRISPER-Cas9 technology work by delivering the Cas9 nuclease complexed with a synthetic guided RNA (gRNA) into a cell, the cell’s genome can be cut at the desired location, allowing existing genes to be removed and/or new ones added (Hendel et al., 2015). The CRISPER-Cas9 is a gene editing technique, simply designed, with high efficiency at relatively low cost, and it has replaced other old techniques for gene manipulation. Recently, Chang et al. (2016) showed that CRISPER-Cas9 targeted miRNA-17, miRNA-200c and miRNA-141, repressed their activity in human colon cancer cell lines HCT116 and HT-29. Furthermore, *in vivo* targeting was effective for at least a month (Chang et al., 2016). However, off-target mutagenesis and effects of a single miRNA on various gene targets are the limitations to the use of this modern technology specifically in brain disorders like prion diseases (Hendel et al., 2015). Beside CRISPER-Cas9 there is another technique known as anti-miRNA oligonucleotides (AMOs), used to control cellular mechanics. These are synthetically designed molecules, used to neutralize the miRNAs function in cells for desired responses (**Figure 4**). By controlling miRNAs that regulate mRNAs expression in the cells, AMOs can be used for therapeutic treatment of certain neurodegenerative disorders. AMOs uses the steric-blocking mechanism for the regulation of miRNAs, and hybridization to target miRNAs (Lennox and Behlke, 2011). In order to determine the functionality of certain AMOs, the AMO/miRNA binding expression (transcript concentration) must be measured against the expressions of the isolated miRNA. The direct detection of differing levels of gene expression allows the relationship between AMOs and miRNAs. This can be detected through luciferase activity. AMOs are commonly used for the diseases involving miRNAs such as cancers, muscular diseases, autoimmune disorders, and viruses. Their efficacy in neurological disorders is still unclear, and there are some major constraints on to the delivery of AMO’s in the brain. Some of these constraints are the poor *in vivo* stability of AMO’s, Inappropriate biodistribution of AMO’s, disruption and saturation of all endogenous RNA machinery after AMO’s therapy and side effects of AMO’s related therapies. Hence, further molecular research is required for their therapeutic use in neurodegenerative disorders.

## Concluding Remarks and Future Directions

Many research groups focus on prion diseases since the discovery of a fatal prion disease in humans known as variant CJD (Somerville et al., 1997; Lezmi et al., 2003). Recently, molecular research increasing the lifespan in different animal models is a promising start toward finding the best possible therapeutic intervention strategy in prion diseases. Still, there is no available therapeutic strategy curing prion-afflicted individuals completely (Castilla et al., 2008). Recently, miRNAs-mediated molecular research in the field of neurodegenerative diseases, such as AD, PD, HD, and prion diseases has made significant progress. Autophagy plays a crucial role in protein misfolding neurodegenerative diseases. Autophagic flux is necessary for the degradation of misfolded/aggregated PrP^Sc^ in prion diseases (Speldewinde et al., 2015), and regulation of autophagic flux via miRNAs may prove beneficial in these diseases, as seen in other neurodegenerative diseases. The role of miRNAs in modulating autophagy is still unclear, and there are some research groups that found beneficial effects of reduced autophagy (Zhang et al., 2016), while on the other hand, some research groups found promotion of autophagic flux as a useful therapeutic strategy to reduce toxic effects of misfolded proteins (Kim et al., 2016). We propose a hypothesis based on previous data in the field of prion-related research, where several groups found autophagic flux as a useful therapeutic strategy toward degradation of misfolded prion proteins, and ultimately reducing the number of damaged organelles (Johnson et al., 2013; Speldewinde et al., 2015). This could be achieved through miRNAs-mediated regulation of autophagy. Some research groups already worked on miRNAs profile of prion disease-infected animals and individuals, but to date, none of the research group focused on miRNAs-mediated regulation of autophagy in prion diseases. Therefore, the starting point would be to identify those miRNAs, which regulate autophagy in prion diseases. Next step after identification of these miRNAs would be upregulation or downregulation of these miRNAs to regulate autophagic flux in prion diseases. Neuroinflammation and neurodegeneration are hallmarks of prion diseases, and discovery of miRNAs that are important for reducing neuroinflammation and neurodegeneration via modulating autophagy could make a breakthrough toward achieving the goal of therapeutic intervention in prion diseases.

## Author Contributions

SS wrote the manuscript. DZ conceived the idea for the study. TH and NS helped with figure preparation. LY critically reviewed the manuscript before final submission.

## Conflict of Interest Statement

The authors declare that the research was conducted in the absence of any commercial or financial relationships that could be construed as a potential conflict of interest. The reviewer ALCC and handling Editor declared their shared affiliation.
